# *In vivo* analysis of protein crowding within the nuclear pore complex in interphase and mitosis

**DOI:** 10.1038/s41598-017-05959-w

**Published:** 2017-07-18

**Authors:** Hide A. Konishi, Suguru Asai, Tomonobu M. Watanabe, Shige H. Yoshimura

**Affiliations:** 1 Laboratory of Plasma Membrane and Nuclear Signaling, Graduate School of Biostudies, Kyoto University, Yoshida Konoe-cho, Sakyo-ku, Kyoto 606-8501 Japan; 2 RIKEN Quantitative Biology Center (QBiC), Suita, Osaka 565-0874 Japan

## Abstract

The central channel of the nuclear pore complex (NPC) is occupied by non-structured polypeptides with a high content of Phe-Gly (FG) motifs. This protein-rich environment functions as an entropic barrier that prevents the passage of molecules, as well as the binding sites for karyopherins, to regulate macromolecular traffic between the nucleoplasm and the cytoplasm. In this study, we expressed individual Nups fused with a crowding-sensitive probe (GimRET) to determine the spatial distribution of protein-rich domains within the central channel *in vivo*, and characterize the properties of the entropic barrier. Analyses of the probe signal revealed that the central channel contains two protein-rich domains at both the nucleoplasmic and cytoplasmic peripheries, and a less-crowded central cavity. Karyopherins and other soluble proteins are not the constituents of the protein-rich domains. The time-lapse observation of the post-mitotic reassembly process also revealed how individual protein-rich domains are constructed by a sequential assembly of nucleoporins.

## Introduction

The nuclear pore complex (NPC) is located in the nuclear envelope and functions as the sole gate for macromolecular trafficking between the cytoplasm and the nucleoplasm in eukaryotic cells^1^. The NPC comprises several copies of more than 30 different protein subunits called nucleoporins (Nups)^2^. The central channel of the NPC functions as a molecular sieve-like diffusion barrier for accurate cargo selection, which allows only small molecules (typically less than 30 kDa in mass or smaller than 5 nm in diameter) to diffuse passively through the pore^3^. Molecules larger than this are excluded by the barrier unless they associate with the appropriate nuclear transport receptors (NTRs) such as karyopherins (importins and exportins)^2, 3^. The molecular mechanism by which karyopherins travel through the barrier despite of their large molecular size is not fully understood; however, a repetitive amphiphilic helical structure (HEAT repeat) has been demonstrated to play an important role in overcoming such a crowded NPC barrier^4–6^.

The selective barrier of the nuclear pore complex is composed mainly of Nups, which contain intrinsically disordered (non-structured) regions (IDRs) with phenylalanine-glycine (FG) motifs (FG-Nups)^7^. Similar to other IDRs (for a review see ref. 8), FG-Nups form self-assembled structures *in vitro* via non-specific interactions between polypeptides as well as via hydrophobic interactions between Phe residues. Individual Nups have different amino acid compositions; therefore, the entire channel is believed to comprise of different types of such self-assembled protein-rich structures (e.g. hydrogels^9^ and liquid-droplets^10^). Although the detailed spatial distribution of such structures in the NPC is not fully understood, the protein-rich environments made by FG-Nups are important for the barrier function. The diffusion rate of karyopherins in the NPC depends clearly on the protein concentration of the channel *in vitro* ^11^, as well as *in silico* ^12^.

In contrast to the analyses *in vitro*, the structure of the central channel *in vivo* is poorly understood. The properties of the selective barrier have been deduced mainly from so-called *in vitro* transport assays, in which the plasma membrane of cultured cells are permeabilized and then incubated with fluorescently-labeled transport complexes (e.g. karyopherin and its cargo)^13, 14^. Analyzing the kinetics of the labeled cargo in and out of the nucleus could reveal the function of transport receptors and the involvement of individual Nups in the transport event. Recently developed single-molecule observation techniques, whether *in vivo* or *in vitro*, also visualized the behavior of a single fluorescently labeled cargo with high spatial resolution^15^ and revealed an energy landscape of the central channel. However, since the central channel of the NPC works as a large entropic barrier to prevent the passage of molecules, as well as an energy basin which attracts NTRs by a number of FG-motifs, the behavior of the transport complex can be interpreted as a sum of these two factors. Therefore, it is very important to obtain the information of entropic and enthalpic factors of the central channel separately.

In this study, we focused on the entropic barrier effect of the NPC and aimed to determine the distribution of protein crowding in the central channel. To this end, we used a fluorescent protein-based probe, which is able to detect protein crowding in a living cell^16^. The probe was fused with individual FG-Nups and expressed in a cultured cell. Analyses of the probe signal revealed protein crowding around individual Nups, spatiotemporal distribution of such protein-rich domains within the NPC, and how they are assembled in the post-mitotic assembly process.

## Results

### GimRET probe is sensitive to protein solution of FG-Nups

Several fluorescent protein-based probes are sensitive to highly concentrated protein solutions (protein crowding) and are able to evaluate local protein crowding^16, 17^. GimRET was first developed by fusing the cyan fluorescent protein (CFP) with a glycine-inserted yellow fluorescent protein (YFP1G)^18^. The fluorescence intensity of YFP1G decreases as the surrounding concentration of protein increases (Figure S1)^16^. There is a clear inverse relationship between the protein concentration and the fluorescence intensity of YFP1G. The dynamic range of the probe is more than 200 mg/mL (Figure S1). Note that such sensitivity is not observed for any other fluorescent proteins including CFP^16^, which allows us to use it as reference signal in the fluorescence imaging. By utilizing FRET (Förster resonance energy transfer) between CFP (donor) and YFP1G (acceptor), protein crowding could be evaluated by taking the signal intensity ratio between the acceptor and the donor; GimRET probe, when existed in higher protein crowding, has lower acceptor/donor signal ratio, due to decreased fluorescence intensity of the acceptor (YFP1G)^16^.

**Figure 1:**
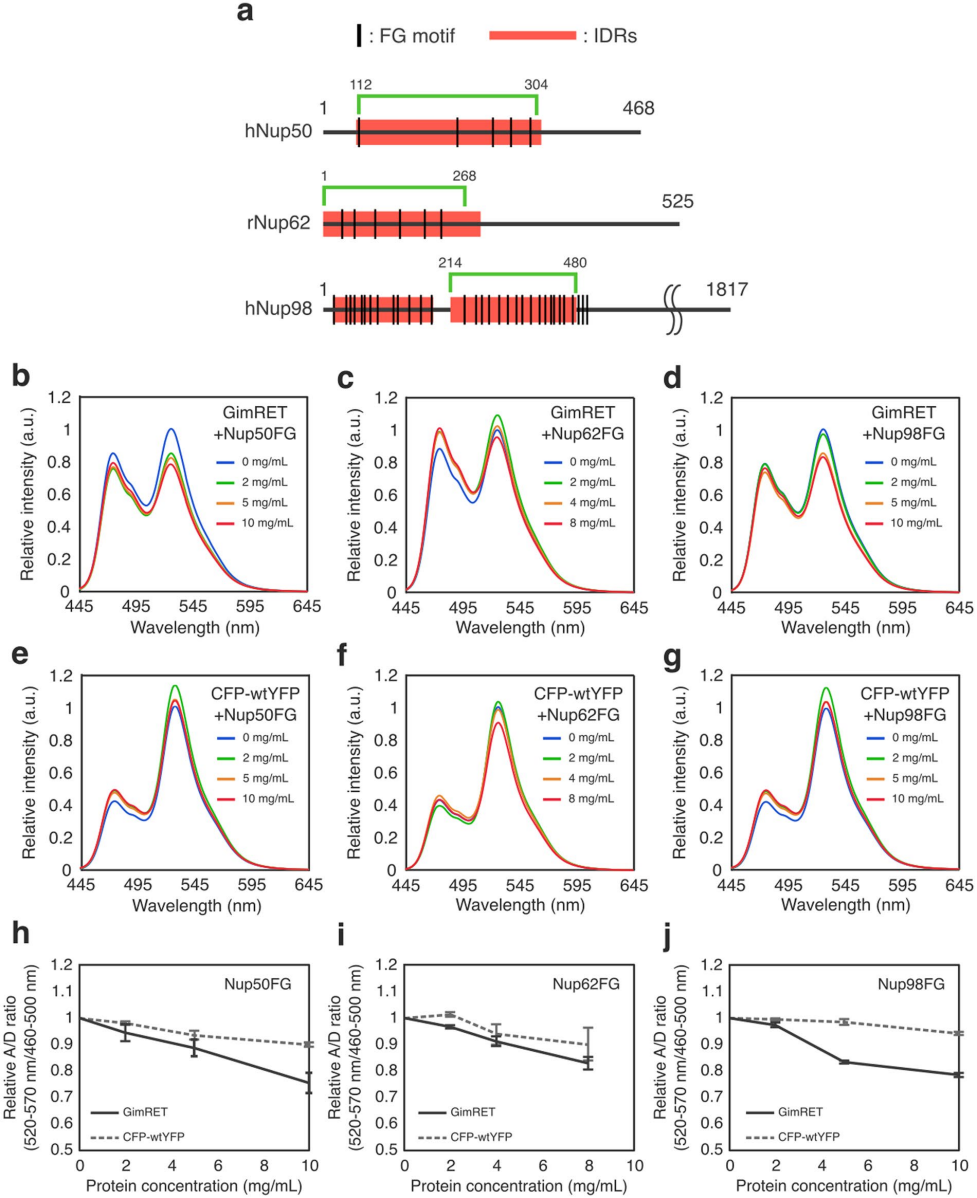
GimRET senses crowding of Nups. (**a**) Schematic representation of FG-fragments used in the probe assay. The regions for FG-fragments used in *in vitro* crowding assay are shown in green. Intrinsically disordered regions (IDRs) predicted by PONDR algorithm are shown with red bar. UniProtKB accession numbers for sequence prediction: Q9UKX7, hNup50; P17955, rNup62; P52948, hNup98. (**b–g**) Fluorescence spectra of purified GimRET (**b–d**) and CFP-wtYFP (**e–g**) in the presence of different concentrations of FG-fragments (Nups50 (**b** and **e**), Nup62 (**c** and **f**), and Nup98 (**d** and **g**)). Emission spectra with excitation wavelengths of 433 nm are shown. Concentrations of Nups were as follows: 0 mg/mL, blue; 2 mg/mL, green; 5 mg/mL, orange; 10 mg/mL (8 mg/mL for Nup62FG), red. (**h–j**) Acceptor/donor (A/D) ratios from GimRET (solid line) and CFP-wtYFP (dotted line) are plotted against Nup concentration, Nup50FG (**h**), Nup62FG (**i**) and Nup98FG (**j**). Data are presented as mean ± SD, from three independent experiments.

We first examined whether GimRET could sense highly concentrated FG-Nups in solution. Protein fragments containing FG motifs (Nup50FG, Nup62FG, and Nup98FG, Fig. 1a) were expressed in bacterial cells and purified. The fluorescence spectrum of GimRET was measured in the presence of a high-concentration of these purified FG fragments. As shown in Fig. 1, the addition of increasing amounts of FG fragments affected the fluorescence spectrum of GimRET significantly, in a concentration-dependent manner (Fig. 1b–d,h–j). The fluorescence spectrum of the CFP-wtYFP probe, in which YFP1G was replaced by wild-type YFP, was not severely affected by the same FG fragments at any of the concentrations tested (Fig. 1e–j). There was no relationship between the number of FG motifs and the effect on the acceptor/donor signal ratio (Figure S2), which suggested that the probe is not specific for the phenylalanine residues. Hereafter, we defined the acceptor/donor (A/D) signal ratio as the “probe signal” and a decrease in the probe signal as an indicator of protein crowding.

### *In vivo* analysis of protein crowding in the NPC

**Figure 2:**
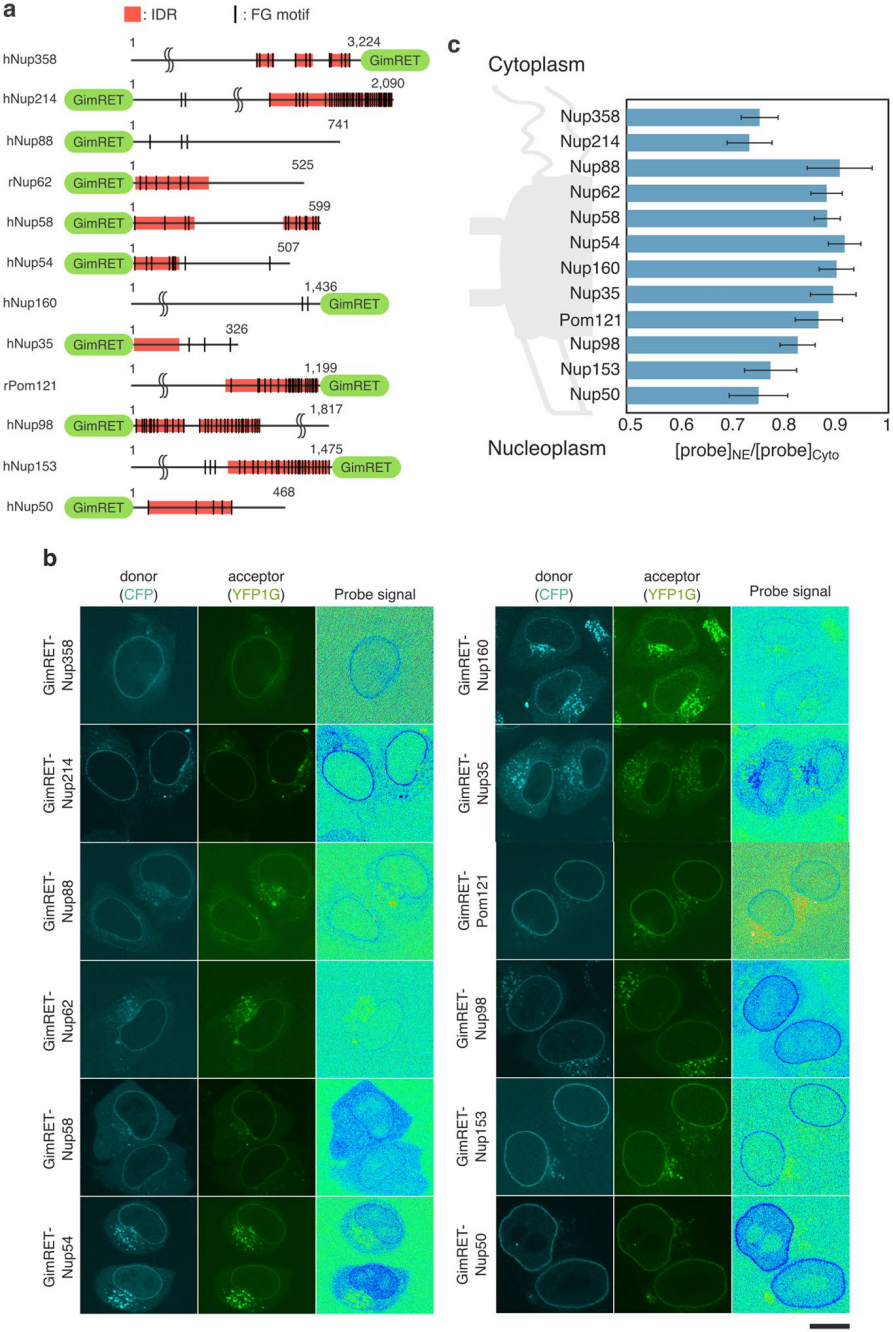
The NPC contains two protein-rich domains in both peripheries. (**a**) Schematic representation of GimRET-fused Nups used in this study. Intrinsically disordered regions (IDRs) are predicted by PONDR algorithm and shown with red bar. FG-motif is indicated with a vertical line. The position of the GimRET probe is also depicted at either end of the polypeptide. UniProtKB accession numbers used for sequence prediction: P49792, hNup358; P35658, hNup214; Q99567, hNup88; P17955, rNup62; Q9BVL2, hNup58; Q7Z3B4, hNup54; Q12769, hNup160; Q8NFH5, hNup35; P52591, rPom121; P52948, hNup98; P49790, hNup153; Q9UKX7, hNup50. (**b**) Fluorescence imaging of HeLa cells expressing GimRET-fused Nups. The sample was excited by 435 nm, and the donor (CFP, 460–500 nm, left), and acceptor (YFP1G, 520–570 nm, middle) images were captured. The ratio (acceptor/donor) image is also shown (right). Scale bar: 20 μm. (**c**) A summary of probe signals from 12 different GimRET-fused Nups. The probe signal (acceptor/donor) at the nuclear envelope ([probe]_NE_) was measured in individual cells and presented as a relative value to that in the cytoplasm ([probe]_Cyto_) (also see Figure S3d). This means that protein crowding in each part of the NPC was evaluated as a relative value to that in the cytoplasm. Data are aligned from top to bottom according to the approximate localization along the pore axis from the cytoplasmic to the nucleoplasmic ends as previously described^19^. Data are presented as mean ± SD from more than 20 different cells. Some Nups showed some punctae, which represented crowding (Fig. 2, Nup214 and Nup35). These crowded punctae can be considered the artifacts by the overexpression because GimRET-fused another type of target proteins related to cytoplasmic aggregates showed similar crowding (data not shown).

Protein crowding within the NPC was examined by expressing GimRET-fused Nups in HeLa cells, and then analyzing the probe signal by ratiometric fluorescence imaging. A number of previous studies demonstrated that fluorescent protein-fused Nups can be incorporated into the NPC without affecting its function, and can be used to estimate the copy number of individual Nups^7^. The GimRET probe was fused to either the amino or carboxyl terminus of each FG-Nup, depending on which is closer to the FG-rich IDR (Fig. 2a). Figure 2b shows fluorescence images of GimRET-fused Nups. All of the probe-fused Nups tested were localized properly in the nuclear envelope, as well as in the cytoplasm. The image of the probe signal was obtained by taking the ratio of acceptor over donor signals. As shown in Fig. 2b, the probe signal from the GimRET-fused Nup was significantly lower in the nuclear envelope compared with that in the cytoplasm. To eliminate the possibility that exogenous GimRET-fused Nups were overloaded to the NPC and caused non-physiological crowding, we examined the relationship between the signal intensity of GimRET-Nups in the NPC (YFP1G signal) and the probe signal. As shown in Figure S3a–c, the probe signal is independent of the expression and localization levels of the GimRET-fused proteins. Furthermore, to confirm the decrease of the probe signal is due to the property of YFP1G, the CFP-wtYFP probe (insensitive to protein crowding, Fig. 1) was also fused with Nups and expressed in HeLa cells. As shown in Figure S4, the probe signal in the nuclear envelope was almost the same as that in the cytoplasm, indicating that the reduction in the probe signal in the nuclear envelope is specific to GimRET.

The probe signal in the nuclear envelope ([probe]_NE_) region was analyzed and represented as the relative value to that in the cytoplasm ([probe]_Cyto_) in the same image (the detail of the image analysis is described in the legend of Fig. 2 and Figure S3d). This is based on the assumption that the IDRs, to which the GimRET probe is fused, do not form any specific secondary structures in the cytoplasm. As summarized in Fig. 2c, each Nup showed slightly but significantly different degrees of protein crowding. When the probe signals from individual Nups were plotted along with the spatial distribution of each Nup in the channel axis^19^, several distinct protein-rich domains could be identified: the FG-Nups located at the periphery of the pore (Nups50, 98, 153, 214, and 358) showed larger reductions in the probe signal (~25%, highly crowded), whereas those located in the central cavity (Nups54, 58, and 62) showed smaller reductions (~10%, less crowded). Nups in the scaffold also showed a large decrease in the probe signal, but to a lesser extent compared with that of the peripheral Nups. These results implied that the NPC contains at least two protein-rich domains: one located in the cytoplasmic periphery that is mainly composed of Nups214 and 358; and the other located on the nucleoplasmic side, and comprising Nups50, 98, and 153, leaving the central cavity less crowded.

### Effect of transport receptors and inhibitors on the protein-rich domains in the NPC

Previous studies demonstrated that the NPC contains a significant amount (~1 MDa) of NTRs and their cargos^20–22^, suggesting that they might be important determinants of protein crowding within the NPC^23^. To examine the effects of NTRs and their cargos on local protein crowding within the NPC, we performed *in vitro* transport assays as previously described^24, 25^. HeLa cells expressing GimRET-fused Nups were first treated with digitonin to permeabilize the cell membrane and remove cytoplasmic proteins. The cells were then incubated with purified recombinant proteins or inhibitors, subjected to microscopic observation, and analyzed as described above.

**Figure 3:**
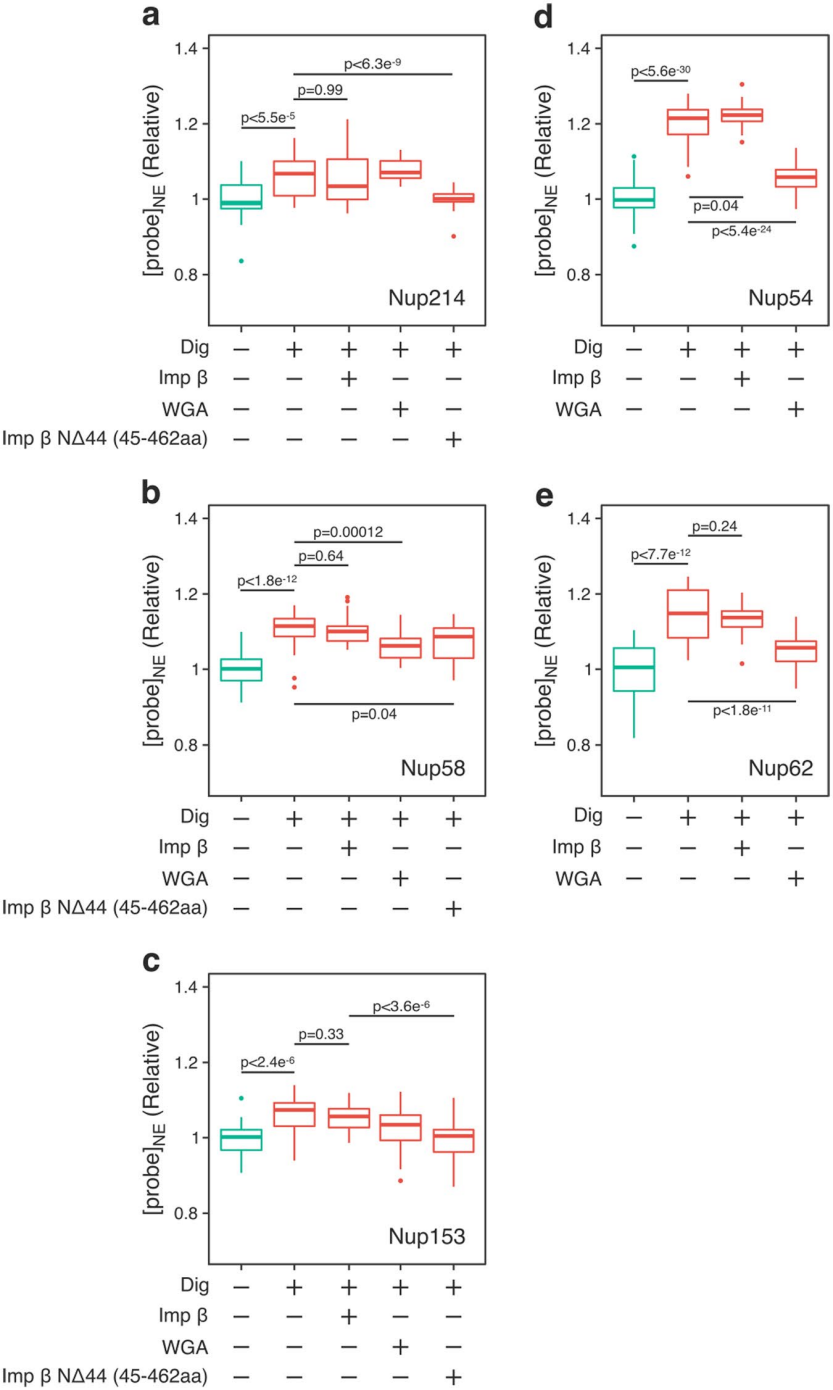
Transport inhibitors affect protein crowding in the NPC. HeLa cells expressing GimRET-fused Nups (214 (**a**), 58 (**b**), 153 (**c**), 54 (**d**) and 62 (**e**)) were treated with digitonin (Dig), followed by wild type of importin β (10 μM), wheat germ agglutinin (WGA, 50 μg/mL) or a fragment of importin β (Imp β N∆44, 45–462aa, 10 μM). The probe signal in the nuclear envelope ([probe]_NE_) after each treatment was analyzed and shown as the relative value to the probe signal of intact cells. Data are presented as 25% and 75% quartiles; median, bold line; outliers, ○, from over 30 different cells. P-values are obtained from two-tailed Welch’s t-test.

Immunostaining with anti-karyopherin β antibody revealed that the digitonin treatment removed endogenous importin β from the NPC by 30% (Figure S5a), suggesting that digitonin treatment removed significant amount of cellular proteins from the NPC. In good agreement with this, the probe signal from GimRET-fused Nups also reduced by digitonin treatment (Fig. 3). Digitonin itself had no effect on the emission spectrum of GimRET (Figure S5b–d). The effect was most pronounced in the central cavity (Nups54, 58, and 62), whereas it had little effect on the peripheral Nups, suggesting that a significant amount of transported molecules occupy the central cavity *in vivo*. This result agreed partially with our finding that the central cavity is plugged by protein-rich domains at both (cytoplasmic and nucleoplasmic) ends, such that the transported proteins tend to accumulate in the central cavity (see *Discussion*).

The addition of purified importin β (10 μM, higher than the physiological concentration, ~3 μM^26^) to digitonin-treated cells increased the amount of importin β in the NPC judged by immunostaining (Figure S5e), but did not affect protein crowding of all the Nups tested. This suggested that importin β itself is not a major component of protein crowding within the central channel. On the other hand, a mutant form of importin β (impβ-NΔ44, 45–462aa), which is known to accumulate in the NPC and inhibit karyopherin-dependent nuclear transport^27^ (Figure S5e), increased crowding in both nucleoplasmic and cytoplasmic peripheries as well as the central cavity (Fig. 3a–c). The same effect was observed with another transport inhibitor, wheat germ agglutinin (WGA)^28^. The addition of WGA after digitonin-treatment increased crowding in the central cavity specifically (Nups54, 58 and 62) (Fig. 3b,d, and e). This is consistent with a previous report that WGA binds to Nups in the central cavity^29^ and inhibits the passage of transport cargo.

**Figure 4:**
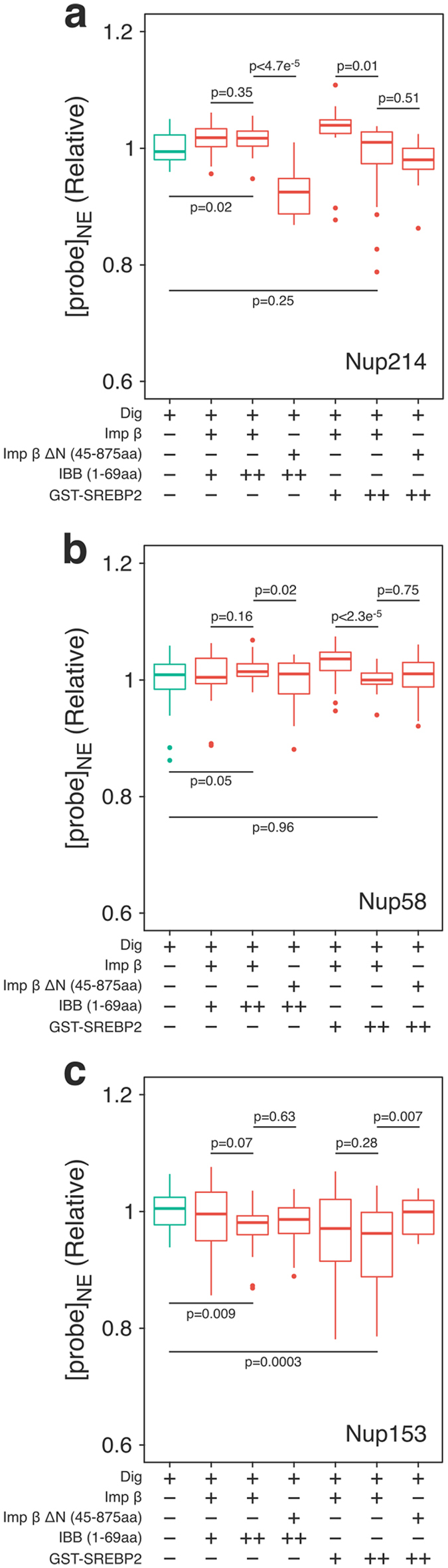
Importin β-dependent nuclear transport affects protein crowding in the NPC. Similar to Fig. 3, HeLa cells expressing GimRET-fused Nups (214 (**a**), 58 (**b**), 153 (**c**)) were treated with digitonin (Dig), and incubated with indicated components of the transport; wild-type of importin β (10 μM), a deletion mutant of importin β (Imp β∆N, 45–875aa, 10 μM), importin β binding domain of importin α (IBB), glutathione S–transferase (GST)–sterol regulatory element–binding protein 2 (SREBP2) (+: 1 μM, ++: 10 μM), together with RanGDP, NTF2 and ATP regeneration system. The probe signal in the nuclear envelope ([probe]_NE_) after incubation was analyzed and shown as the relative value before the incubation. Data are presented as 25% and 75% quartiles; median, bold line; outliers, ○, from over 20 different cells. P-values are obtained from two-tailed Welch’s t-test.

We also tested whether importin β-dependent nuclear transport affects protein crowding in the NPC. Digitonin-treated cells expressing GimRET-Nups were incubated with importin β, cargo, RanGDP, NTF2 and ATP regeneration system^30^. We tested two cargos with different molecular sizes; importin β binding domain of importin α (IBB, ~8 kDa) and glutathione S–transferase–tagged sterol regulatory element–binding protein 2 (GST-SREBP2, ~150 kDa)^31^. Importin β-mediated nuclear transport increased protein crowding around Nup153, but had little effect on Nup58 (Fig. 4c). These results are in good accordance with our *in vivo* measurement of protein crowding, in which nucleoplasmic Nups had higher crowding than the central cavity (Fig. 2). The cargo size (IBB or GST-SREBP2) affected the crowding, but the amount of the cargo had only little effect. Interestingly, crowding around Nup214 was not affected by importin β-dependent transport, implying that import and export events cause protein crowding at different domains within the NPC. When an importin β mutant (Imp β∆N, 45–875aa) which binds to the NPC but cannot bind to RanGTP^27, 32^ was used in this assay, the crowding of Nup214 was increased, implying that the inhibition of cargo release at the nucleoplasmic side can affect the movement of the transport complex at the cytoplasmic side (see *Discussion*).

### Subunit assembly and formation of protein-rich domains in late mitosis

During mitosis, the NPC disassembles into subunits or subcomplexes, and reassembles in the late stages of mitosis^33–35^. Previous studies revealed that individual Nups assemble on anaphase chromosomes in a defined and ordered series of events^35, 36^. We therefore tried to determine how the protein-rich domains observed in interphase NPCs are formed during the post-mitotic reassembly process.

**Figure 5:**
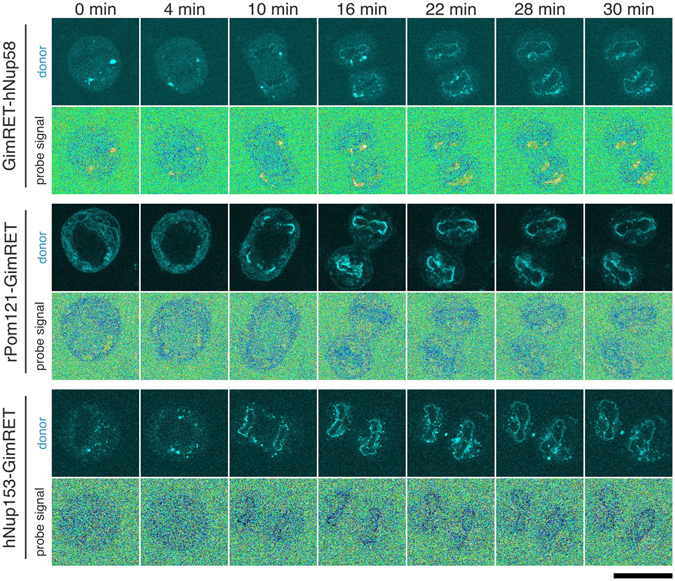
Time-lapse observation of mitotic cells expressing GimRET-fused Nups. HeLa cells expressing GimRET-fused Nups (top, Nup58; middle, Pom121; bottom, Nup153) and mPlum-fused histon H3 were subjected to time-lapse imaging during mitosis. CFP (localization) and probe signals at the indicated time points after anaphase onset are shown. Scale bar: 20 μm.

GimRET-fused Nups were expressed in HeLa cells with mPlum-fused histone H3 as a chromosome marker, and time-lapse observations were carried out from metaphase to early G1 phase. As shown in Figs 5 and S6, the GimRET-fused Nups started to localize on chromosomes in anaphase. We analyzed the following two signals around chromosomes and plotted them against the time after anaphase onset; (i) the CFP signal as an indicator of the localization of the target Nup; and (ii) the probe signal as an indicator of protein crowding around the target Nup, which was also represented as the relative value to that of the cytoplasm of early G1 phase in the same series of images.

**Figure 6:**
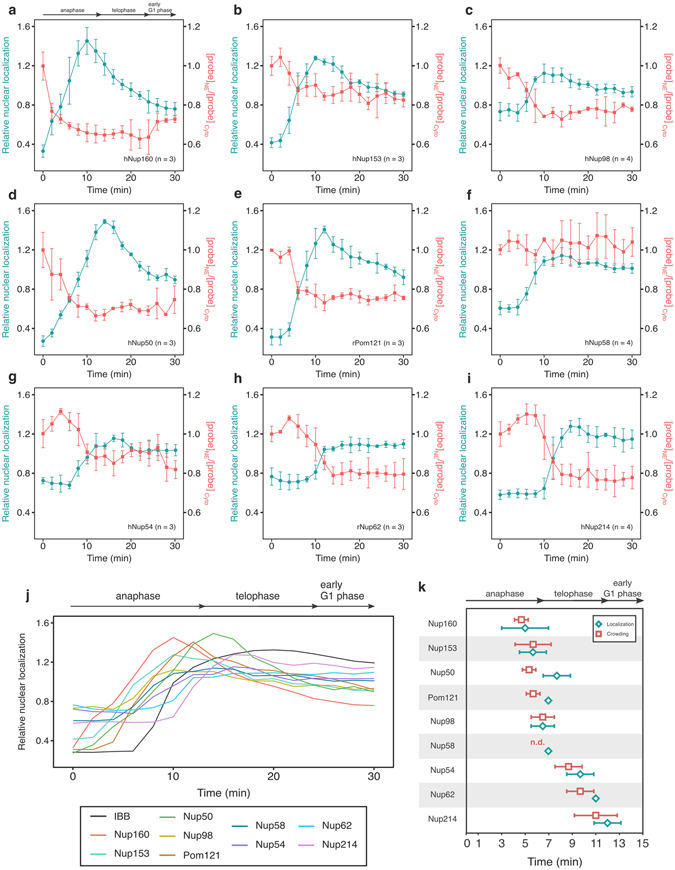
Time-lapse analysis of localization and crowding of Nups during mitosis. (**a–i**) Co-plot of Nup localization and protein crowding. The CFP signal on the chromosome surface was measured as a localization signal, and shown as the relative nuclear localization value as previously reported^59^. The relative localization signal (blue), and the probe signal ([probe]_NE_/[probe]_Cyto_, red) are co-plotted against time after anaphase onset, which was defined by the separation of chromosomes (mPlum-H3). The probe signal is presented as a relative value to that at time 0. The results from Nups 160 (**a**), 153 (**b**), 98 (**c**), 50 (**d**), Pom121 (**e**), Nup58 (**f**), 54 (**g**), 62 (**h**), and 214 (**i**) are shown. Data are represented as mean ± SD, with the number of the cells indicated (**n**) in the upper side of each panel. (**j**) Summary of the localization signals. Relative nuclear localization of CFP signals shown in the panel (**a–i**) are summarized. Representative fluorescence images are shown in Figure S5. The progression of mitosis is indicated at the top of the panel. For visibility of the graph, error bars are not shown here. (**k**) Summary of localization and probe signals during mitosis. The time when the localization signal and probe signal reached half maximum and minimum was extracted from results represented in Fig. 4a–i, and plotted against time after anaphase onset. Data are represented as mean ± SD from more than 3 independent experiments. Peak crowding of Nup58 (n.d) was not plotted because the probe signal did not significantly change during the observation (**f**).

The analysis of the CFP signal revealed a clear relationship between the order of assembly and the position in the NPC, as was demonstrated in previous studies^35, 36^. The NPC is constructed from the nucleoplasmic towards the cytoplasmic region. After the initial assembly of the scaffold subunit (Nup160) (~4 min), FG-Nups present in the nucleoplasmic protein-rich domain (Nups153, 50, and 98) and the membrane-spanning subunit (Pom121) appeared (~6 min) (blue lines, Fig. 6a–e). Nups in the central cavity (Nups54, 58, and 62) followed at 6–10 min (blue lines in Fig. 6f–h), and Nups in the cytoplasmic protein-rich domain (Nup214) finally assemble at the end of anaphase (~13 min) (blue line, Fig. 6i). Note that the Nups in the central cavity (Nups54, 58 and 62) appeared with slightly different timings to each other (Fig. 6j and k), implying that they completely disassemble during mitosis, although they form a stable complex during interphase^29^. The timing of Nup62 assembly was similar to that of Nup214, supporting their direct interaction^29, 37^ (see *Discussion*). It should also be noted that the localization signal intensity of the early-assembling Nups started to decrease during telophase (~10 min after anaphase onset, Fig. 6a–e), whereas that of the other Nups (central cavity and cytoplasmic) remained almost constant after assembly (Fig. 6f–j), implying a dynamic disassembly of early-assembled Nups (see *Discussion*).

Quantification of the probe signal from the same set of the images showed the relationship between the subunit assembly and the formation of protein-rich domains. As shown in Fig. 5, protein crowding formed during late-mitosis was observed in accordance with the localization of Nups ([CFP]_NE_). Note that the probe signal during the reconstruction process was also independent of the localization levels of the GimRET-fused proteins, eliminating the possibility that over-loading of GimRET-Nups to the constructing NPC caused non-physiological protein crowding (Figure S7). The probe signals from early-assembling subunits (Nups160, 153, 50, 98, and Pom121) dropped by ~20% as soon as they appeared on anaphase chromosomes (red lines in Fig. 6a–e). This indicated that the scaffold subunits (Nup160), the membrane-spanning subunit (Pom121), and the nucleoplasmic FG-Nups (153, 50, and 98) form the initial nucleoplasmic protein-rich domain immediately after localization on the chromosome surface. In contrast to the localization signal, which started to decrease in telophase, the probe signal of the early-assembling Nups remained constant after they formed the protein-rich domain (compare blue and red lines in Fig. 6a–e). These observations suggested that a certain amount of the early-assembling Nups fail to form the protein-rich domain after assembly on the chromosome surface and become dissociated (see *Discussion*).

Protein crowding in the central cavity (Nups54, 58, and 62) was relatively small (~5%) in interphase cells (Fig. 2c). However, the probe signal of these Nups in mitosis dropped by 5–20% upon the assembly to the NPC, suggesting that the crowding state of the central cavity largely changes during cell cycle probably due to the property and amount of transported proteins. Notably, the assembly of Nups54, 58, and 62 did not affect protein crowding in the protein-rich domain formed by early-assembled Nups, implying that the central cavity and the nucleoplasmic domain are separated spatially. Protein crowding around Nup 214 increased upon its incorporation into the complex at late anaphase (Fig. 6i), which did not affect protein crowding of other previously assembled Nups. The timings of the assembly and protein crowding of Nup214 were very similar to those of Nup62, implying that their behavior is coordinated in the reassembly process.

## Discussion

In this study, we analyzed the spatial distribution of protein crowding within the central channel of the NPC in live cells. The analysis revealed at least two major protein-rich domains located at the cytoplasmic and nucleoplasmic peripheries of the channel, each of which comprises a distinct set of Nups. As summarized in Fig. 2c, all Nups tested in this study showed higher protein crowding in the NPC than in the cytoplasm, although to different degrees. The GimRET probe was fused to the end of the polypeptide that was close to the FG-rich IDR (except for Nup214, which did not localize in the NPC). Considering that IDRs generally do not form secondary structures, a reduction in the probe signal in the NPC indicates directly that the FG-rich domains form highly crowded microenvironments. The YFP1G protein (the acceptor unit of the GimRET probe) is sensitive to high concentrations of protein and other macromolecules^16^. Although the detailed molecular mechanism of this sensitivity is not fully understood, it can be speculated from its crystal structure that it is sensitive to the hydrophobic surfaces of polypeptides, which disturb the hydration layer around the region where one glycine residue was inserted^16, 18^. The fact that GimRET-fused Nups showed significant reductions in the signal in the NPC indicates that these Nups have close and/or frequent contacts with other protein molecules. GimRET is not sensitive to the number of FG-motifs (Figure S2); therefore, the signal in the NPC directly indicates the density of Nups rather than the amount of crosslinking between Nups.

We found two protein-rich domains at the peripheries of the central channel in intact cells (Fig. 2). This crowding was more (around central cavity: Nups54, 58, and 62) or less (around both protein-rich domains: Nups214 and 153) reduced by digitonin (Fig. 3); therefore, the protein-rich domains at the peripheries are composed mainly of intrinsic structural components of the channel (FG-Nups) and not of soluble NTRs and their cargos. The protein-rich domain composed of FG-Nups should work as a large entropic barrier for transported cargos, as well as a binding site for NTRs. The balance of these entropic losses and enthalpic gains defines the behavior of the NTRs. The existence of two such protein-rich domains at the peripheries of the central channel (Fig. 2) provide a good explanation of the behavior of the transport complex observed in previous studies. Single-molecule fluorescence observation of the NTR-cargo complex during passage through the NPC revealed that the NTR-cargo complex spent most of the transport time at both the cytoplasmic and nucleoplasmic peripheries^38^. Another single-molecule study also revealed that the residence time of the importin α/CAS complex was longer at the cytoplasmic and nucleoplasmic ends (~4.2 ms; cytoplasmic, ~0.9 ms; nucleoplasmic), and shorter in the central part (~0.3 ms)^14^. High spatial-resolution imaging (electron microscopy and super-resolution microscopy) of the NTRs within the NPC revealed preferred distributions of the NTRs at both peripheries of the pore^15, 39, 40^. These results suggested that the nucleoplasmic and cytoplasmic peripheries of the central channel contain a high density of proteins and work as entropic barriers; however, because of their FG-motifs, they also attract the NTRs and works as their binding sites.

In contrast to the peripheral regions, our quantification suggested that the central cavity of the channel is less crowded (Fig. 2). This means that it does not work as an entropic barrier. This correlated with the behavior of the NTRs described above (NTRs do not stay long in the central cavity), and further suggested that the central cavity does not function as a strong binding site for the NTRs^41^. This was in good agreement with the results shown in Fig. 3, in which the addition of purified importin β to the digitonin-treated cells did not change the crowding of the central cavity. This suggested that the NTRs that reached this cavity successfully after passing through either protein (FG)-rich domain (nucleoplasmic or cytoplasmic), are immediately trapped again by either protein (FG)-rich domain. When it is trapped by the same domain (on the same side of the central cavity), the transport is “aborted”, whereas if it is trapped by the opposite domain, the transport is “successful”, as was shown in the previous single-molecule observations^42^.

Our result from *in vitro* transport assay (Fig. 4) also supports this model. Protein crowding around Nup153 was significantly increased upon the addition of the transport complex (importin β and its cargo, in the presence of RanGTP gradient) depending on the size of the cargo (IBB or GST-SREBP2). This may be due to high affinity of importin β to Nup153, which carries a larger number of FG motifs and has the smallest dissociation constant (*K*_D_: 9 ± 25 nM) among FG-Nups^41, 43^. Importin β–cargo complex may spend longest time around Nup153. Interestingly, the crowding around Nup214 was increased when a large amount of the large cargo (GST-SREBP2) was added, and when the cargo release was abolished at the nucleoplasmic side by importin β mutant (imp β Δ44) (Fig. 4), suggesting that retardation of the transport at the nucleoplasmic side of the pore affects the crowding of the cytoplasmic protein-rich domain. Therefore, it might be plausible that cytoplasmic and nucleoplasmic protein-rich domains are two tandemly connected binding sites along the central channel, and when one site is occupied by transport complexes, it will affect the other. In living cells, not only importin β, but also other karyopherin β family proteins and other transport mediators carry their cargos for both directions (inbound and outbound) and occupy most of the binding sites within the NPC.

The effect of WGA on the structure of the central channel, as well as on the transport events, is also explained by our results using the GimRET probe. WGA binds to *O*-linked glycosyl groups and inhibits karyopherin-dependent transport^28^. Although *O*-linked glycosyl groups have been identified in many Nups (Nups54, 58, 62, 98, 153, and 214^28, 44^), WGA binds strongly to the central cavity complex^28, 45, 46^, and reduces the mobility therein^47^. Our results demonstrated that WGA increased the crowding of the central cavity (Nups54, 58 and 62) (Fig. 3b,d and e), indicating that it occupies the cavity spatially. Occupation by WGA excludes the NTRs from the cavity and inhibits them from reaching the protein (FG)-rich domains on the opposite side, resulting in frequent “abortion” of the transport. This was supported by the electron microscopic observation showing that the gold-labeled nucleoplasmin is excluded from the central cavity after WGA treatment^48^. Furthermore, importin β N∆44 (45–462aa)^27^, also increased crowding in the central channel (Fig. 3a–c). Interestingly, the degree of increase of crowding was prominent in both cytoplasmic (Nup214) and nucleoplasmic (Nup153) side. This result reinforces our results, which demonstrated that the central cavity is not a major binding site for importin β even if it’s a mutant of importin β.

Our time-lapse observations of mitotic cells expressing GimRET-fused Nups enabled simultaneous observation of Nup localization with the formation of the protein-rich domains around each Nup during the post-mitotic reassembly process. This information is very useful to understand the process of subunit assembly during post-mitotic NPC reconstruction. The results are summarized as follows. i) Nups comprising the central channel are assembled from the nucleoplasmic towards the cytoplasmic sides on anaphase chromosomes. ii) The formation of the nucleoplasmic protein-rich domain (Nups50, 153, and 98) is completed before the assembly of the central cavity (<10 min after anaphase onset). iii) Significant amounts of nucleoplasmic Nups dissociate from the initial complex of the NPC, without forming the protein-rich domain. iv) The cytoplasmic protein-rich domain is assembled during late anaphase, independently of the nucleoplasmic domain.

Careful analysis of the localization signal revealed several important features of the mitotic assembly of Nups. Nups in the central cavity (Nups54, 58 and 62) are known to form a stable heteromeric complex during interphase^49^. Our results shown in Fig. 4 indicated that individual Nups are recruited to the chromosome surface at different times between 6–10 min after anaphase onset, suggesting that they disassemble during mitosis, and reassemble in the post-mitotic reconstruction process. Phosphorylation might regulate the interaction among these Nups^50, 51^. Nup62 assembled just before Nup214 (Fig. 6h,i, and k). This agreed with a recent result from pull-down assay in which Nsp1 (the yeast homolog of human Nup62) formed a complex with Nup159 (the yeast homolog of human Nup214) and Nup82 (the yeast homolog of human Nup88) to build the cytoplasmic ring^52^. The behavior of Nup62 and Nup214 in our time-lapse observation may suggest that Nup62 recruits Nup214 to form the cytoplasmic ring structure.

**Figure 7:**
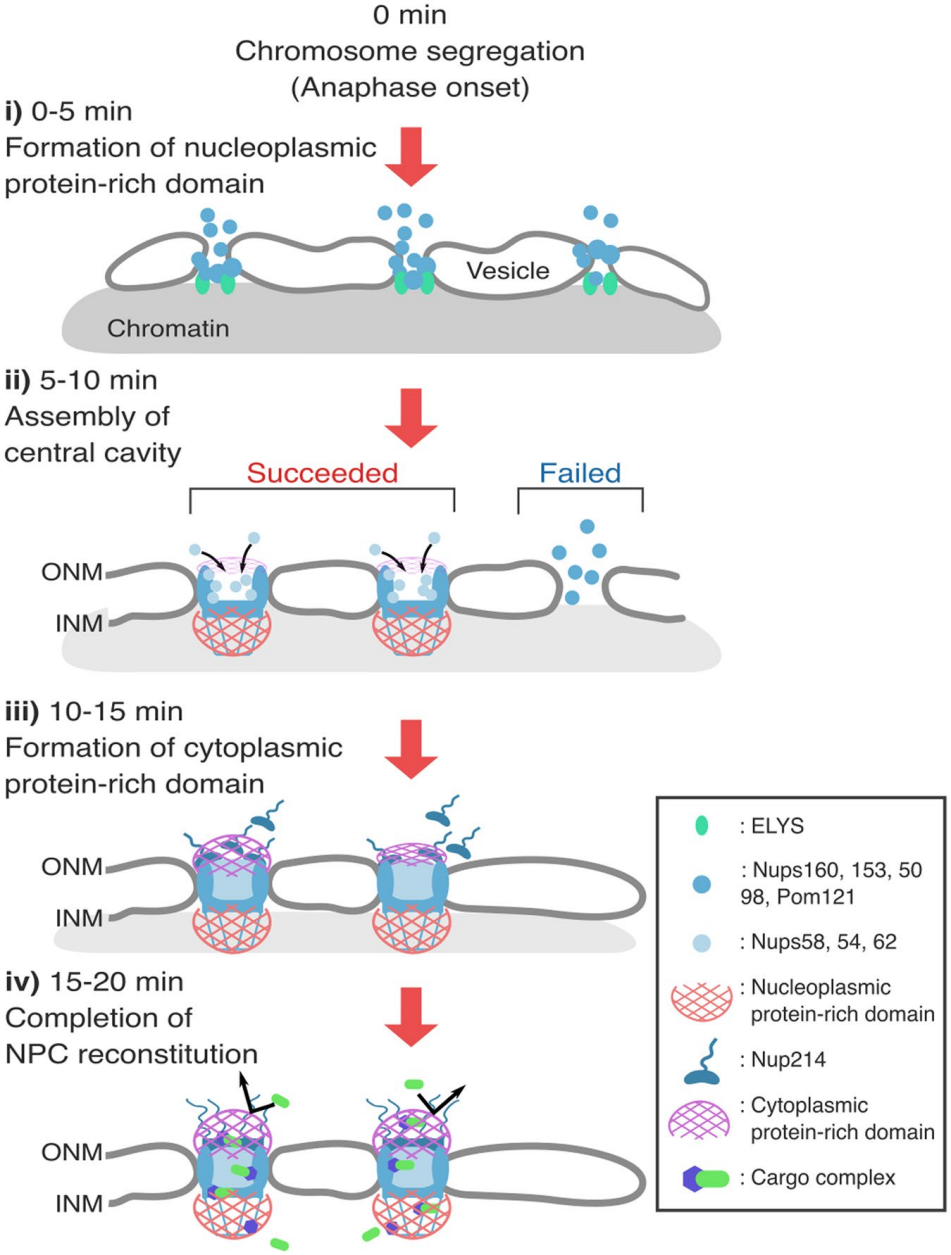
Schematic illustrations of the protein-rich domains during post-mitotic NPC reconstitution process. Schematic model of the post-mitotic reassembly process of the NPC. i) NPC reconstitution starts around 0–5 min after the onset of anaphase. After the initial binding of ELYS to the chromosome, scaffold and nucleoplasmic Nups start to assemble^60, 61^, and immediately form the nucleoplasmic protein-rich domain (red). ii) When it is properly assembled, it recruits Nups in the central cavity. There are also a significant number of miss-assembled complexes, which will dissociate from the chromosome surface and diffuse into the cytoplasm before assembling the central cavity Nups. iii) Following the assembly of the central cavity, the cytoplasmic Nups bind to the complex, and immediately form the cytoplasmic protein-rich domain. Nup98, which localizes to both ends of the pore, might also contribute to the formation of the cytoplasmic protein-rich phase (not shown here). iv) After 15–20 min, the reconstitution of the entire NPC with two distinct protein-rich phases is completed. INM: inner nuclear membrane; ONM: outer nuclear membrane.

It is intriguing that the localization of early-assembling Nups on the chromosome gradually increased after the onset of anaphase, but subsequently decreased in telophase until the early G1 phase (Fig. 6a–e). This marked reduction in the signal is specific to the early-assembling Nups, and was not observed for other Nups (Fig. 6f–i). The reduction in the signal during telophase was not caused by expansion of the chromosome surface area accompanying chromosome decondensation, because the time-lapse plot of the relative chromosome surface area did not match this reduction pattern (Figure S8). Another more plausible possibility is that a certain early-assembling Nups dissociate from the chromosome surface during telophase (Fig. 7). It is possible that during the early assembly process, some FG-Nups might form a protein-rich domain successfully, but others may fail. Successfully assembled Nups are then able to recruit the central cavity subunits and continue the reconstitution process, whereas unsuccessfully assembled Nups will eventually dissociate/detach from the complex and diffuse back into the cytoplasmic pool (Fig. 7ii). It should be noted that the probe signal of early-assembling Nups increased when they assembled on the chromosome, but remained constant when the localization signal decreased, suggesting that only successfully assembled Nups, but not unsuccessful ones, form the protein-rich domain. The fact that the assembly of Nups54, 58, and 62 coincided with the dissociation of early-assembling Nups implied that the coordinated formation of the nucleoplasmic protein-rich domain acts as a checkpoint before proceeding to assembly of the central cavity (Fig. 7).

In conclusion, our study on the quantification of protein crowding within the NPC in living cells demonstrated that the formation of local protein-rich domains, which are possibly caused by the self-assembly property of IDR-containing polypeptides with distinct sets of Nups, is associated with the ordered assembly of the NPC. These findings provide a clue to understand the mechanism of how thousands of polypeptides are assembled and coordinated into a functional NPC during the cell cycle, and these also demonstrated a useful technique to evaluate local protein crowding in a living cell.

## Materials and Methods

### DNA construction and protein purification

The construction of DNA fragments encoding GimRET and CFP-wtYFP was described previously^16^. They were sub-cloned into the pET28-b(+) vector (Novagen). FG-fragments of hNup50 (aa 112–304), rNup62 (aa 1–268) and hNup98 (aa 214–480) (see also Figure S2) were amplified by PCR from the full-length cDNA and sub-cloned into pET28-b(+). His_6_-tagged CFP-wtYFP and GimRET were expressed in *Escherichia coli* (BL21-CodonPlus(DE3)-RIL, Agilent Technologies, Wilmington, DE) by induction with 0.5 mM IPTG at 20 °C for 16 hours, and then purified by Ni-NTA agarose beads (QIAGEN). His_6_-tagged FG fragments were purified using Ni-NTA agarose beads (QIAGEN) under denaturing conditions (8 M Urea) with a pH gradient^9, 53, 54^. His_6_-tagged proteins (RanGDP, NTF2) were also purified using Ni-NTA agarose beads (QIAGEN) according to the manufacturer’s protocol and dialyzed in transport buffer (20 mM HEPES, pH 7.3, 110 mM potassium acetate, 5 mM sodium acetate, 2 mM magnesium acetate, and 1 mM EGTA). GST-tagged IBB, SREBP2, and mouse importin β as well as mutants of importin β were expressed in *E. coli* and purified as described previously^55, 56^, and dialyzed against transport buffer.

Purified FG-fragments were dialyzed against 0.05 or 0.1% (v/v) trifluoroacetic acid (Nacalai Tesque) for 24 h, lyophilized, and then dissolved in a small volume (~100 μL) of 250 mM HEPES. The pH of the protein solution was adjusted to between 7.5 and 8.4. The final concentration of the protein solution was determined using a Bio-Rad *DC* ^TM^ Protein Assay Reagent (BIO-RAD) or Coomassie brilliant blue (CBB) staining, with bovine serum albumin (BSA) as a standard.

### Fluorescence spectra measurement

The fluorescence spectra of purified GimRET and CFP-wtYFP were measured using a spectrophotometer (FP-8300, JASCO) with a one-drop measurement unit (SAF-851). BSA (SIGMA) and lysozyme (Nacalai Tesque) were dissolved and dialyzed against 250 mM HEPES (pH 7.5–8.4) and stored at 4 °C as stock solutions. Purified fluorescent protein (GimRET or CFP-wtYFP) was mixed with proteins at various concentrations (0–250 mg/mL for BSA and lysozyme, 0–10 mg/mL for FG solutions), and subjected to spectroscopic measurements. As previously described^57^, the acceptor/donor (A/D) ratio was calculated as the total value of the fluorescence intensities between 520–570 nm divided by that between 460–500 nm.

### Expression of GimRET-fused Nups in HeLa cells

DNA regions encoding EGFP in pEGFP-N1 and pEGFP-C1 vectors (Invitrogen) were replaced by that of CFP-wtYFP or GimRET. Full-length cDNAs encoding hNups50, 54, and 58, were amplified by PCR from a cDNA pool of HeLa cells. These fragments and cDNAs encoding rNup62 and hNup153 (kind gifts from K. Ullman, University of UTAH), hNup98 (a kind gift from T. Haraguchi, Advanced ICT research center), hNup160 (a kind gift from B. Fahrenkrog, University of Basel), hNup214, 358, and rPom121 (kind gifts from J. Ellenberg, EMBL), were sub-cloned into the CFP-wtYFP and GimRET vectors. Plasmid DNA was introduced into HeLa cells, which were cultured in Dulbecco’s modified Eagle’s medium (DMEM, SIGMA) supplemented with 10% fetal bovine serum (FBS), using a lipofection reagent (Effectene, QIAGEN). Microscopic observations were performed 26 h after transfection.

### Microscopic observation and image analysis

HeLa cells expressing CFP-wtYFP- or GimRET-fused Nups were washed three times with phosphate buffered saline (PBS; SIGMA) and fixed with 4% (w/v) paraformaldehyde (SIGMA) at room temperature for 20 minutes. Semi-permeabilized cells were prepared by digitonin (50 μg/mL) treatment in transport buffer, as described previously^4, 58^. After digitonin treatment, the cells were washed three times with transport buffer and incubated for 15 min at room temperature with a 1 mg/mL BSA solution (Roche), and subsequently incubated for 15 min at room temperature with 10 or 100 μM purified importin β in TB. For WGA treatment, 50 μg/mL of WGA (SIGMA) was applied after digitonin treatment, the cells were incubated for 15 min at room temperature, and then supplemented with BSA or purified importin β. Finally, the cells were fixed with 4% (w/v) paraformaldehyde at room temperature (20–25 °C). For examining the effect of nuclear transport, digitonin-treated cells were incubated with a transport mixture containing 10 μM importin β (wild type or a mutant (Imp β∆N), 25 μM His_6_-RanGDP, 40 μM His_6_-NTF2, 1 or 10 μM of cargo (importin β-binding domain of importin α (IBB (1–69aa)) or glutathione S–transferase (GST)–sterol regulatory element–binding protein 2 (SREBP2)), and ATP regeneration system (1 mM ATP, 5 mM creatine phosphate and 200 U/ml creatine phosphokinase) at room temperature for 10 minutes. The cells were then fixed with 4% paraformaldehyde at room temperature for 20 minutes and subjected to microscopic observation.

Microscopy was performed using FV1200 confocal laser-scanning microscope (Olympus) with a 60 × NA1.35 objective lens. The excitation wavelength was 433 nm, and the emission wavelength was between 460 to 500 nm for the CFP channel, and 520 to 570 nm for the FRET channel. Signal intensities of the nuclear envelope, chromosome rim, and cytoplasm were quantified using MetaMorph (Molecular Devices) software.

### Immunostaining for digitonin-treated HeLa cells

HeLa cells were washed three times with TB and permeabilized as described above. After the digitonin treatment, cells were fixed with 4% (w/v) paraformaldehyde (SIGMA) and immunostained with anti-karyopherin antibody (BD Transduction Laboratories) and fluorescein isothiocyanate-conjugated anti-mouse IgG (Cappel Laboratories) as described in the previous report^58^. Purified importin β was added before the fixation, then incubated, and subjected to microscopic observation as described above.

### Time-Lapse imaging of mitotic cells

GimRET-fused Nups or importin β were co-expressed with mPlum (Clontech)-fused histone H3 in HeLa cells cultured in DMEM (SIGMA) supplemented with 10% FBS. Before the microscopic observations, the culture medium was replaced with phenol red-free DMEM (SIGMA) supplemented with 10% FBS and 8 μM l-glutamine. The stage-top chamber (Tokai-hit) was filled with moisture using distilled water and 5% CO_2_, and was maintained at 37 °C. Images were captured every 2 min until the cell entered the early G1 phase. Signal intensity at chromosome rim was measured manually using MetaMorph (Molecular Devices) software.

## Electronic supplementary material

Supplemental information
