# Supplementary material for: In vivo analysis of protein crowding within the nuclear pore complex in interphase and mitosis
Source: Sci Rep. 2017 Jul 18;7:5709. doi: 10.1038/s41598-017-05959-w (PMC5515885; doi:10.1038/s41598-017-05959-w)
Supplement: Supplementary file 1 — Supplemental information [file 41598_2017_5959_MOESM1_ESM.pdf]

Title:

In vivo analysis of protein crowding within the nuclear pore complex in interphase and mitosis

Author affiliation:

Hide A. Konishi[1], Suguru Asai[1], Tomonobu M. Watanabe[2] and Shige H. Yoshimura[1*]

[1]Laboratory of Plasma Membrane and Nuclear Signaling, Graduate School of Biostudies, Kyoto University, Yoshida Konoe-cho, Sakyo-ku, Kyoto 606-8501, Japan

[2]RIKEN Quantitative Biology Center (QBiC), Suita, Osaka 565-0874, Japan

Supplemental information

Supplemental figure legends

Figure S1 Supplemental fluorescence spectra analyses of CFP-wtYFP probes in several protein solutions.

(a–h) Fluorescence spectra of GimRET (a, b, e, and f) and CFP-wtYFP (c, d, g, and h) in other protein solutions. Emission spectra in response to excitation by 433 nm (a–d) and by 513 nm (e–h) are shown. (i) Acceptor/donor (A/D) ratios of the CFP-wtYFP probe (red, ■) and GimRET (blue) in the presence of increasing concentrations of BSA. Data are normalized by the signal in the absence of BSA. Data were obtained from three independent measurements and were normalized to that in the absence of crowder.

Figure S2 The probe signal is independent of the number of FG-motifs

Relationship between the number of FG motifs and the probe signal. Relative acceptor/donor (A/D) ratio of GimRET in a 10 mg/mL solution of FG-Nup. Data are presented as mean ± SD, obtained from three independent experiments. The maximum concentration of Nup62FG was 8 mg/mL.

Figure S3 The probe signal is independent of the amount of the probe in the NPC

(a) GimRET was expressed in HeLa cells. The probe signal (acceptor/donor ratio) was obtained by the excitation light of 433 nm as described in Figure 2, and YFP1G signal was obtained by the excitation light of 513 nm. The relationship between these two signals (probe and YFP1G) was plotted for all pixels in the image (512 x 512). The probe signal is independent of the YPF1G signal in the range of 500-2000 of YFP1G signal. We used this range of the intensity for all analyses in this study. Similar analyses were performed with the cells expressing GimRET-fused hNup58 (b), and GimRET-fused hNup214 (c). The probe signal was plotted against YFP1G signal from the pixels in nuclear envelope (NE) and cytoplasm (Cyto). The probe signal from GimRET-hNup58 (y-axis) showed similar distribution in the cytoplasm and in the nuclear envelope, although the YFP1G signal (x-axis) was stronger in the nuclear envelope than in the cytoplasm. In contrast, the distributions of the probe signal of GimRET-hNup214 were different between cytoplasm and nuclear envelope. (d) Methodology of image analysis in this study. As a representative, HeLa cells expressing GimRET-fused Nup58 are shown here. The sample was excited by 445 nm, and the donor CFP (left) and acceptor (YFP1G, middle) images were captured. The probe signal ([probe]) is defined as the

ratio of acceptor signal ([acceptor]) and donor signal ([donor]); [probe] = [acceptor]/[donor]. The probe image (right) was obtained by dividing acceptor image by donor image and presented in a rainbow scale. To evaluate protein crowding of the NPC, the probe signal in the nuclear envelope ($[probe]_{NE}$) was divided by the probe signal in the cytoplasm ($[probe]_{Cyto}$) for each cell (also see main text and the legend of Figure 2).

Figure S4 Wild-type YFP does not respond to the protein crowding in the NPC

Fluorescence images of CFP-wtYFP-fused and GimRET-fused Nups214, 58, and 50, representing the cytoplasmic, central cavity, and nucleoplasmic side, respectively. Images from the CFP channel (460–500 nm, left), FRET channel (520–570 nm, middle), and the probe image (right) are shown. Images were taken as described in Figure 2a. Scale bar: 20 μm.

Figure S5. Effect of cytoplasmic and other non-Nups factors on protein crowding in the NPC

(a) Quantification of endogenous importin β in the NPC. Intact and digitonin-treated HeLa cells were prepared as described in Figure 3, and subjected to immunofluorescence microscopy using anti-importin β antibody (BD Transduction Laboratories). Fluorescence signals in the cytoplasm (cyto), nuclear envelope (NE) and nucleoplasm (Nuc) were quantified, summarized, and shown as the relative value to the signal in the NE of intact cells. (b–d) The effect of digitonin and WGA on the fluorescence spectra of GimRET. Fluorescence spectra of GimRET solutions containing digitonin or WGA were obtained by 433 nm excitation (b) and 513 nm excitation (c). Signals were normalized to the maximum value of the control sample, and summarized in (d). Data in (d) are presented as mean ± SD, obtained from three independent experiments. (e) Quantification of endogenous and exogenous importin β in the NPC. Digitonin-treated HeLa cells were incubated with 10 μM of wild-type importin β (WT) or a fragment of importin β (imp β NΔ44) and was subjected to immunofluorescence microscopy as described in (a). Fluorescence signals in the nuclear envelope (NE) were quantified, summarized, and shown as the relative value to the signal in the NE of intact cells shown in (a). Data are presented as 25% and 75% quartiles; median, bold line; outliers, ○, from over 40 different cells.

Figure S6 Time-lapse observation of localization signals for GimRET-fused Nups in mitosis

Similar to figure 5, HeLa cells expressing GimRET-fused Nups, importin β, mPlum-fused histone H3, and mCherry-fused with IBB were subjected to time-lapse imaging during mitosis. Respective fluorescence signals at the indicated time points after anaphase onset are shown. Scale bar: 20 μm.

Figure S7 Characterization of the probe signal in mitotic cells

(a and b) Relationship between the probe signal and localization signal (YFP1G) in the nuclear envelope of the cells expressing GimRET-fused hNup58 (a), and expressing GimRET-fused hNup153 (b) are shown at the time point 10 min after the anaphase onset. Similar to Figure S3, the probe signal was plotted against the localization signal intensity (YFP1G) for the pixels in the nuclear envelope (NE) and cytoplasm (Cyto).

Figure S8 Relationship between chromosome surface area and localization signal

From the same data set shown in Figure 5 and S6, a relative chromosome surface area (■, purple) was determined and plotted against the time after anaphase onset, together with the relative nuclear localization signal (solid, Nup160; dashed, Nup153). Data is presented as the mean ± SD, obtained from 6 different cells expressing mPlum-fused histone H3.

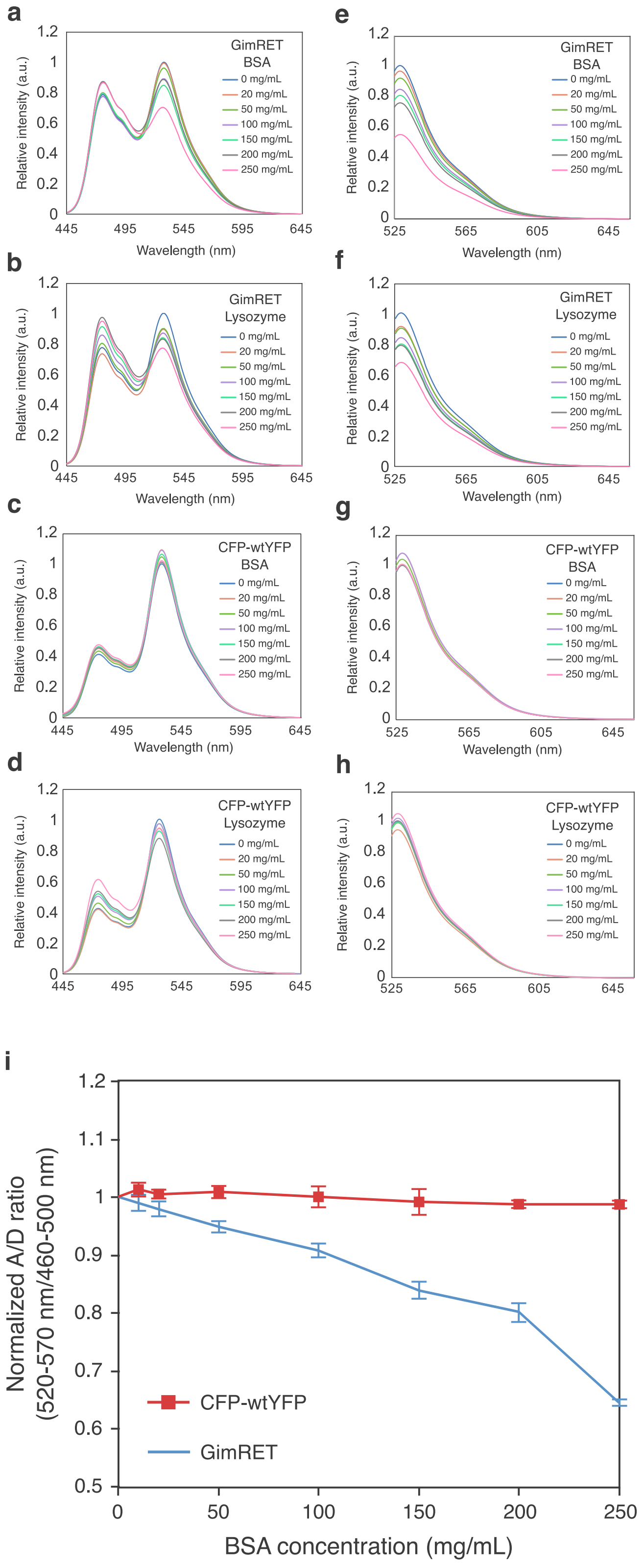

HA. Konishi et al, Figure S1

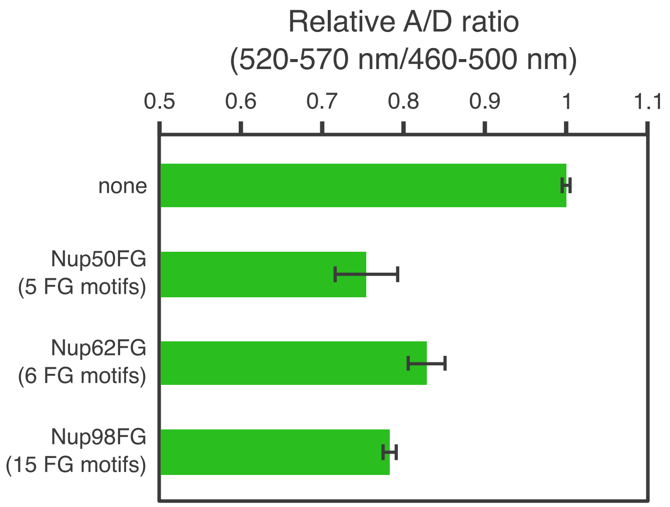

HA. Konishi et al, Figure S2

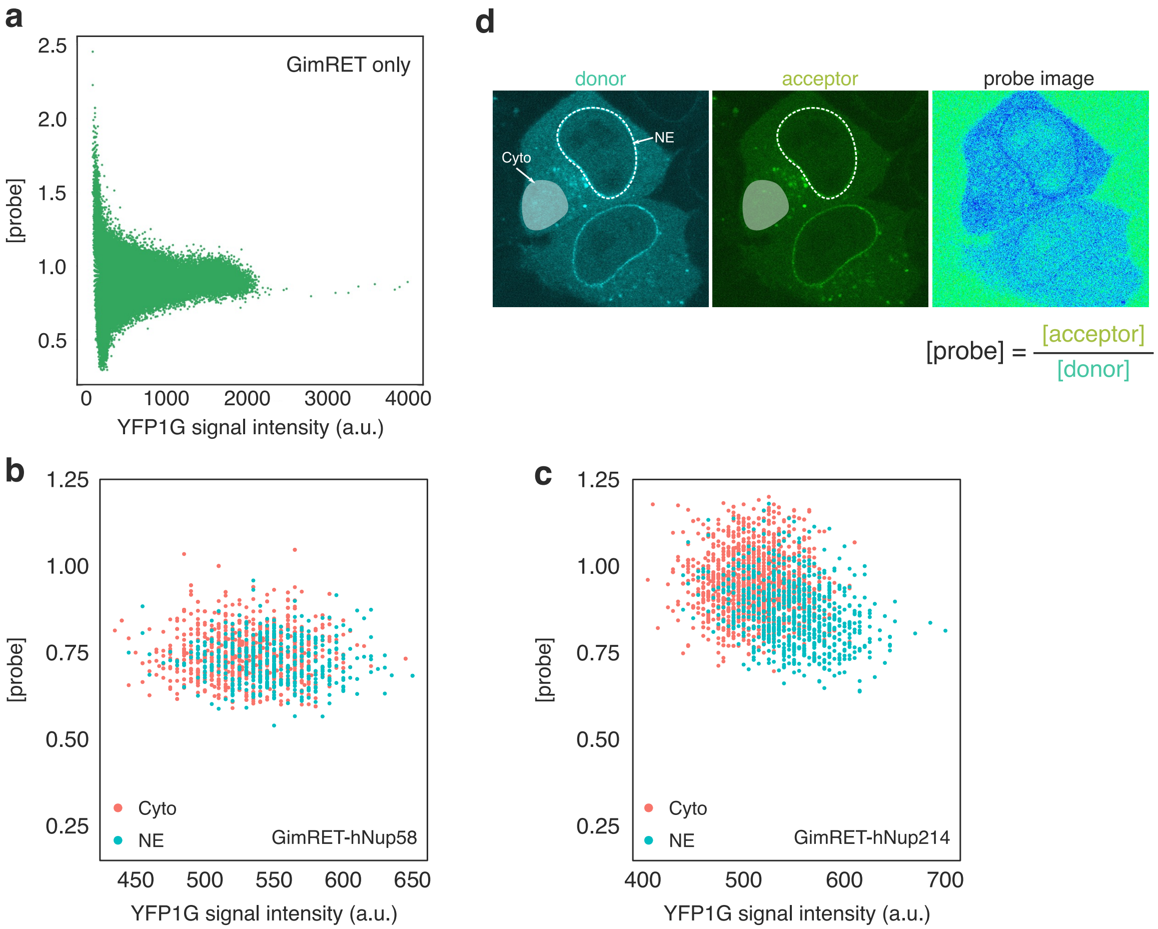

HA. Konishi et al, Figure S3

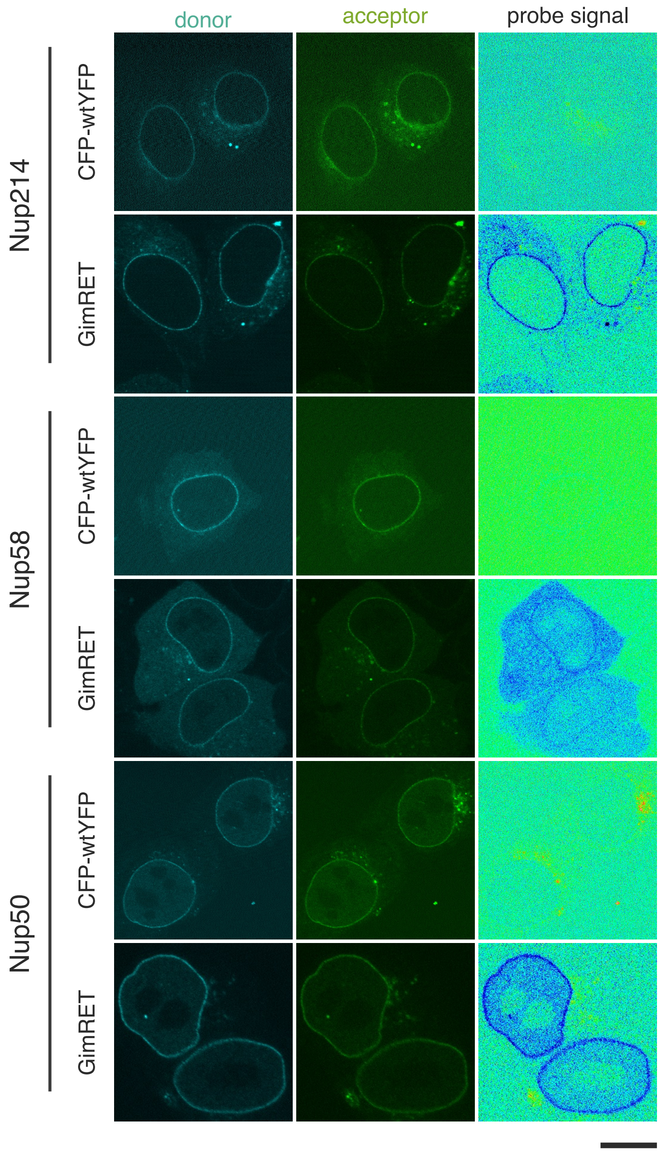

HA. Konishi et al, Figure S4

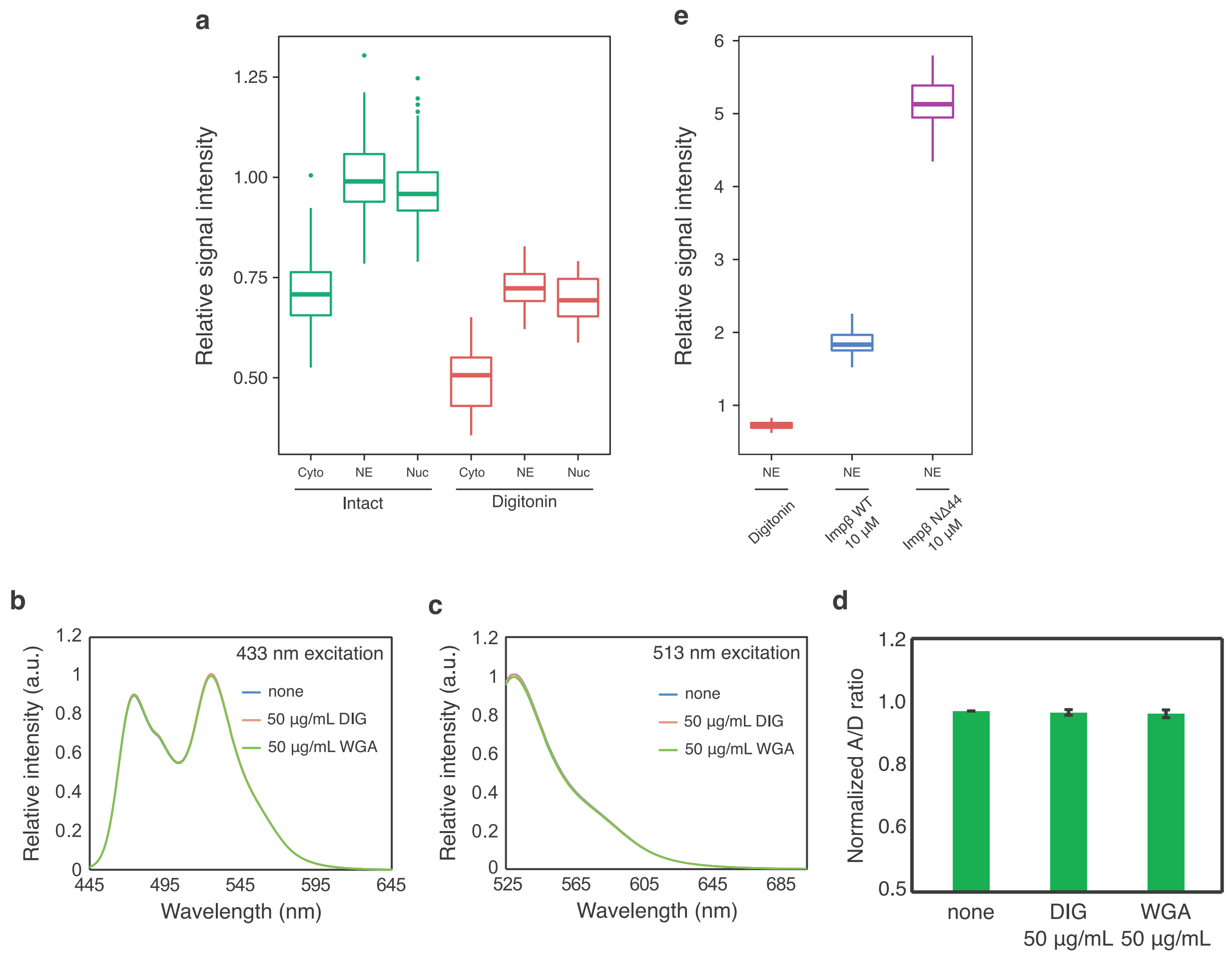

HA. Konishi et al, Figure S5

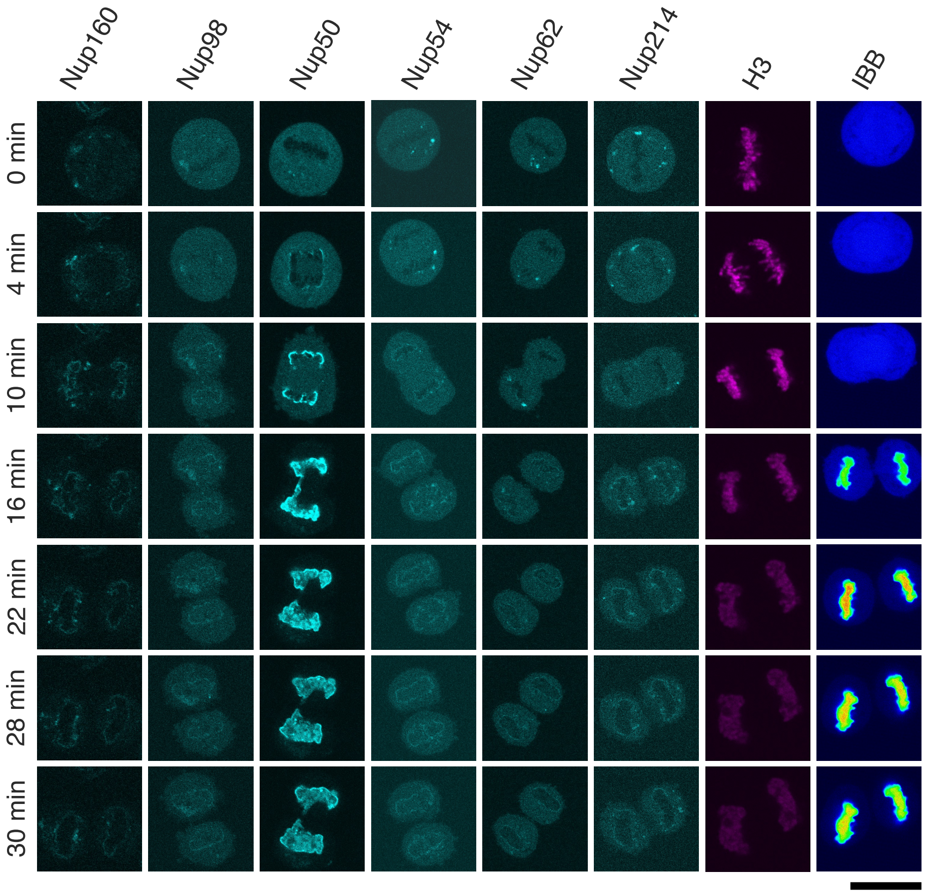

HA. Konishi et al, Figure S6

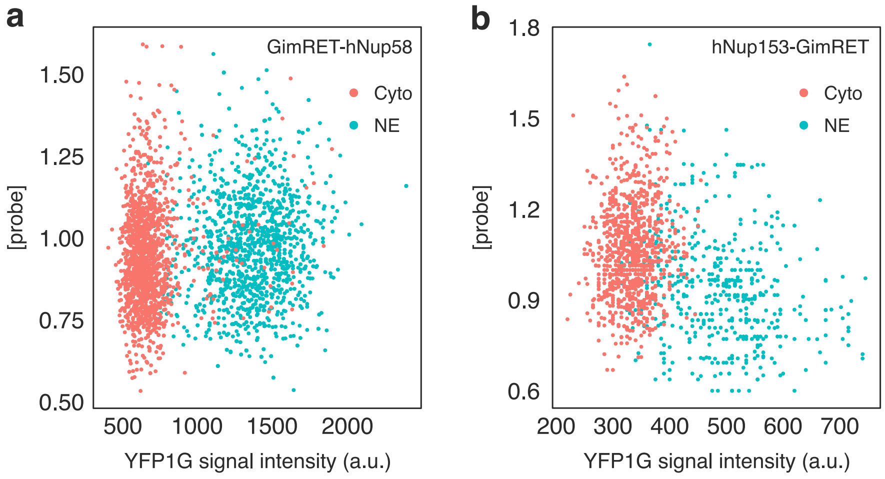

HA. Konishi et al, Figure S7

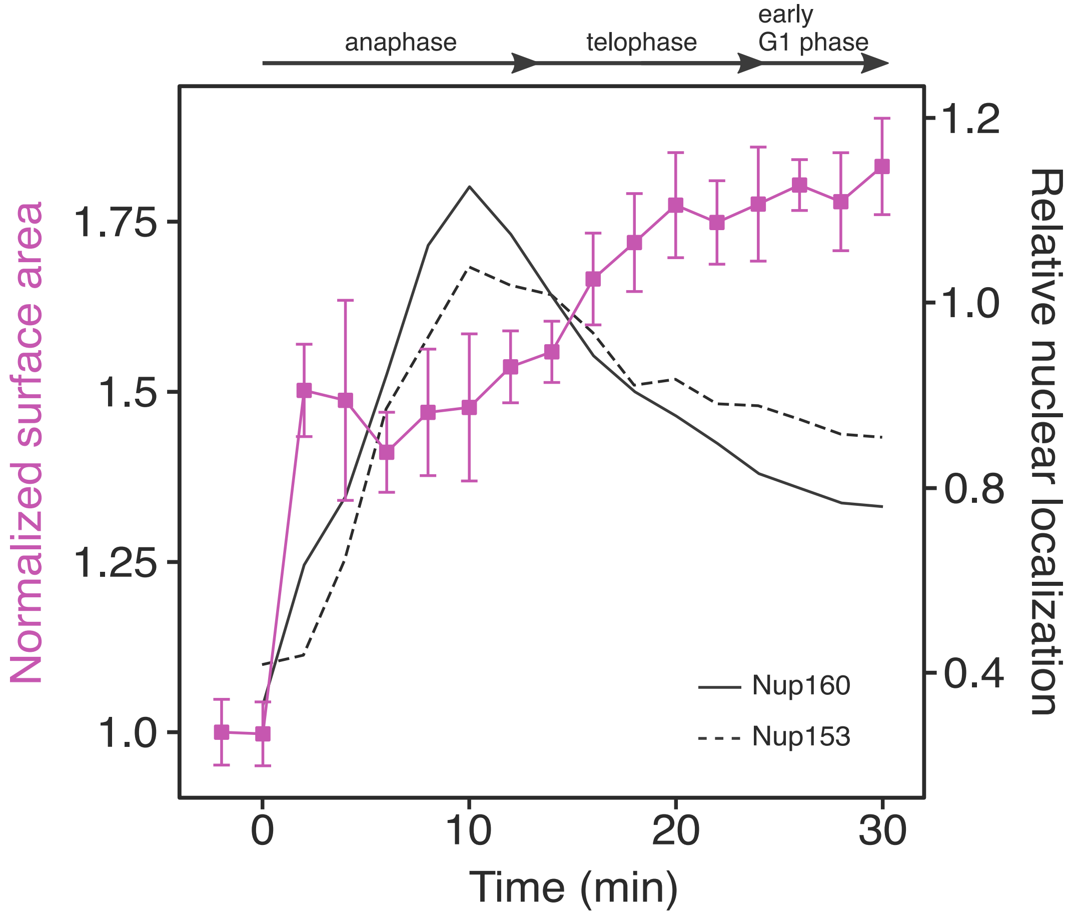

HA. Konishi et al, Figure S8
